# A Standard Protocol for the Clinical Management of Suicidal Thoughts and Behavior: Implications for the Suicide Prevention Narrative

**DOI:** 10.3389/fpsyt.2022.929305

**Published:** 2022-07-12

**Authors:** M. David Rudd, Craig J. Bryan, David A. Jobes, Seth Feuerstein, David Conley

**Affiliations:** ^1^Department of Psychology, University of Memphis, Memphis, TN, United States; ^2^Department of Psychiatry and Behavioral Health, The Ohio State University College of Medicine, Columbus, OH, United States; ^3^Department of Psychology, The Catholic University of America, Washington, DC, United States; ^4^Department of Psychiatry, College of Medicine, Yale University, New Haven, CT, United States; ^5^One More Day, Kuna, ID, United States

**Keywords:** suicide, randomized controlled trials, standard protocol, treatment, public health narrative, risk assessment

## Abstract

The last several decades have witnessed growing and converging evidence from randomized controlled trials (RCT’s) that an identifiable set of simple clinical management strategies are effective for those at risk for suicidal thinking and/or suicide attempts. The current article offers a brief review of clinical strategies supported by RCT’s targeting suicidality as “commonalities of treatments that work” and related recommendations for use in the delivery of care for suicidal individuals in generic fashion, regardless of any particular treatment, theoretical orientation, or intervention perspective. The article includes eight recommendations that can be easily adapted across the full range of clinical contexts, institutional settings, and delivery systems, recommendations that help frame a broader clinical narrative for suicide prevention. Recommendations cut across five identifiable domains or clinical strategies for the delivery of care: (1) informed consent discussion that identifies risks of opting out of care and emphasizes the importance of shared responsibility and a collaborative process, (2) an explanatory model that emphasizes the importance of individual self-management skills and targeting the causes of suicide rather than describing suicidality as a function of mental illness, (3) the importance of proactively identifying barriers to care and engaging in targeted problem-solving to facilitate treatment adherence, (4) a proactive and specific plan for management of future suicidal episodes, and (5) reinforcing the importance of taking steps to safeguard lethal means and facilitate safe storage of firearms.

## Introduction

Over the past several decades, suicide-related research and, of particular importance, randomized controlled trials (RCT’s) from across the globe targeting the treatment and reduction of suicidal thinking and suicide attempts have expanded markedly (1–3), creating an important resource to inform day-to-day clinical care. Results from RCT’s provide a unique opportunity to identify what works with individuals who are suicidal in samples representing a broad range of Axis I-II diagnoses with considerable comorbidity, and what has potential for wider clinical application. Risk of death from suicide is similar to the risks inherent in other medical problems, for example, cardiac death or cancer malignancy. Essential to effective treatment is identifying and targeting the underlying causes. RCT’s provide scientific support for treatment approaches that do just that, effectively identify and treat the underlying causes of suicidal thinking and behavior, independent of the specific psychiatric diagnosis(es) (4).

Jobes and Chalker (5) recently argued that there is a growing body of empirical research to support targeted interventions for those at risk for suicide. But these authors caution that such efforts should be sensitive to the broad array of existing theoretical orientations, different clinical perspectives, and various treatment contexts and settings. Accordingly, they stress the need for a greater focus on clinical interventions and treatments that are optimally matched to identified subgroups of those experiencing suicidal thoughts and engaging in suicidal behaviors.

This article takes another step forward in the evaluation, distillation, and application of available scientific findings. As a complement to meta-analytic studies (2, 3), Rudd and Perez-Munoz (4) identified “commonalities of treatments/clinical interventions that work,” providing a review of common elements in effective RCT’s to date, but fell short of offering specific recommendations for clinical practice. Commonalities were identified by a review and analysis of the treatment/intervention components articulated by the authors of each empirically supported and effective RCT. In short, each RCT identified the mechanisms of action for the targeted treatment/intervention. The points of intersection and overlap of those elements were identified across all RCT’s demonstrated to be effective at reducing suicidal thoughts and/or attempts. Rudd and Perez-Munoz (4) provide a detailed review and list of all RCT’s reviewed (in an expansive table), including study inclusion/exclusion criteria, interventions/treatments tested, follow-up period, outcomes, and significance of subsequent impact. The reader is referred to the table for the specific listing of RCT’s reviewed and specific examples across each domain summarized below. For The current article offers an important extension of this work, providing actionable clinical recommendations for day to day delivery of services with suicidal individuals.

In contrast to previous contributions, like the recommendations offered by the National Action Alliance for Suicide Prevention Zero Suicide Model (6) or the Suicide Prevention Australia Standards for Quality Improvement (7), we focus specifically on the scientific foundation for clinical care created by RCT’s targeting suicidal ideation and behaviors. Accordingly, we offer recommendations regarding the use of simple and effective clinical strategies that can be applied in flexible fashion, regardless of a particular treatment orientation, perspective, or clinical context. These recommendations provide a specific and important empirically supported foundation for clinical practice, regardless of context, one that will hopefully facilitate scientific inquiry essential to clinical practice. Progress in clinical suicidology and subsequent translation to clinical practice is incremental. The integration of proven clinical strategies across clinical contexts will help move that incremental process forward. Additionally, the identified clinical strategies can be used as foundational elements to help shape and inform clinical suicide prevention narratives. The current public health approach is, arguably, disproportionately focused on screening and related risk assessment rather than effective intervention, treatment, and ongoing clinical management of suicide risk.

There is a growing convergence of empirical evidence from RCT’s supporting the effectiveness and use of a limited collection of clinical management strategies with patients who are suicidal, cutting across a broad range of Axis I-II diagnoses with considerable comorbidity (4, 8). Our identified strategies are thus trans-diagnostic and are neither theoretically bound nor clinical-context dependent (i.e., inpatient, outpatient, residential, intensive outpatient, primary care, or emergency department). As a result, the strategies can be integrated into the delivery of effective clinical care regardless of the clinician’s particular theoretical orientation, existing practice approach, treatment timeframe, or treatment setting. These strategies can be organized into a standard clinical management protocol appropriate for broad application, building on the substantive work aggregated by the Suicide Prevention Resource Center (SPRC). Naturally, there is some variability in how strategies might be applied in clinical specialty settings in comparison to, for example, primary care or an emergency department. As Rudd and Munoz-Perez (4) noted, these clinical strategies can most accurately be described as identifiable “commonalities of treatments that work” with individuals who are suicidal. Although each of these strategies has strong empirical support and are in active clinical use across a wide array of settings, to date these strategies have not been organized and integrated into a standard, generic clinical management protocol. Moreover, these findings have yet to be strategically leveraged to help shape narratives focusing on clinical suicide prevention policy and procedures.

A standard clinical management protocol equips the practicing clinician with a solid scientific foundation and framework for working with patients who are suicidal, utilizing strategies demonstrated to be safe and improve clinical outcomes, including reductions in both suicidal thinking and suicide attempts. It is important to emphasize that each of the strategies are conceptualized as interventions requiring action on the part of the clinician, and not simple guiding or underlying principles. In short, the clinician is actively engaged in delivery of the elements summarized in each domain. Available RCT evidence to date supports clinical recommendations that cut across five critical domains: (1) an informed consent process and initial dialogue with people who are suicidal that shares the identified risks of opting out of care and the importance of shared responsibility as part of an effective collaborative treatment process, (2) an explanatory model that helps a patient understand their suicidality such that they can use tailored self-management skills that target the causes of suicide (rather than conceptualizing suicidality merely as a symptom of mental illness and/or psychiatric diagnosis), (3) a proactive approach to identifying and overcoming barriers to care helping facilitate overall treatment adherence, (4) development of a specific plan for successful management of future suicidal episodes, and (5) the importance of taking steps to safeguard lethal means, and in the United States specifically this means a particular emphasis on the safe storage of firearms. All of these recommendations coalesce around a single, central theme that can best be described as the essential importance of recognizing and utilizing a suicide-focused treatment that directly addresses the problems and causes of suicide, routinely monitors patient progress toward these goals, and proactively integrates proven strategies to facilitate patient safety.

The development and application of a standard clinical management protocol has two important advantages for practicing clinicians struggling with how best to integrate scientific advances into day-to-day clinical care with high-risk suicidal individuals. First, and arguably most important, it allows for the integration of clinical strategies demonstrated to be safe and effective for suicidal individuals. Second, utilization of empirically supported strategies also helps build individual self-management skills, improve self-efficacy, facilitate hope, reduce related symptoms, and fuel a wish to live. Arguably, each of the five domains summarized actively engage the patient in clinical activity that facilitates overall self-awareness, self-management, and emotion regulation.

This article is not intended to be a comprehensive review of what is a broad, deep, and rapidly expanding literature base in clinical suicidology. Rather, it is a distillation of critical areas of convergence in the RCT literature that have practical and important relevance for day-to-day clinical care of suicidal individuals across identified domains, with specific recommendations on what and how to implement effective clinical strategies. Although the precise mechanisms of action underlying effective interventions and treatments remain unclear, converging data suggests the simple strategies summarized inspire hope and help develop individual self-management skill that serve a positive and potentially life-saving purpose. In recognition of the converging nature of RCT findings to date and the identified commonalities of treatments that work despite differences in terminology and related jargon, the clinical recommendations offered below are generic in nature and not specific to a given treatment approach or trademarked intervention.

Naturally, the recommendations offered are not static in nature. Rather, they need to continue to grow and evolve as RCT outcome data becomes available. Accordingly, the recommendations will undoubtedly need to be revisited, revised and likely expanded in the coming years. Regardless, they provide a starting point for how to integrate available scientific findings into day to day clinical practice with suicidal individuals.

## Informed Consent and the Initial Dialogue With Individuals Who Are Suicidal Addressing Risks, Personal Responsibility, Collaborative Care, and Treatment Hesitancy

Informed consent is a universal expectation the cuts across all mental health disciplines and is not unique to care to with individuals who are suicidal. Informed consent expectations are articulated in ethics guidelines and licensing boards rules of practice shaping the delivery of clinical care regardless of the severity of suicide risk (9). The full range of issues relevant to risks specific to the treatment of those presenting with suicidality (i.e., risk for suicide, suicide attempts, and suicidal thinking) have been raised and discussed in detail elsewhere and will not be repeated here [e.g., (10)]. Ethics guidelines and licensing boards rules of practice are clear in identifying a professional obligation to share risk information with those considering or engaging in clinical care, including the risks of various treatment alternatives, along with the nature of risk when a decision is made to opt out of care.

A unique commonality from RCT’s targeting suicidality and from the perspective of a standard protocol is that effective treatments not only identify suicide-specific risks, but also include a clear statement about the importance of personal responsibility as part of a collaborative process in treatment, along with the specific expectations of the suicidal individual during care, including the possible integration of family and loved ones into the process from the outset. All those involved in the clinical care process should recognize and accurately understand suicide-specific risks from the start. As noted previously, this is an actual clinical intervention not just a guiding principle, it is an active discussion with the patient, not simply inclusion of text in an informed consent document. Effective RCT’s actively engaged patients in discussions targeting not just suicide-specific risks, but also the importance of personal responsibility and how that translates to specific tasks and skills in clinical care.

From the outset, treatments that work emphasize that the process is collaborative by nature, the importance of personal responsibility, and that embracing both help facilitate treatment adherence essential to effective care and successful outcomes. As noted in a separate recommendation below, this is coupled with a commitment to be proactive in identifying and overcoming barriers to care, rather than simply waiting for problems to emerge that ultimately increase the probability of withdrawal from treatment. There are a range of therapeutic approaches and perspectives in the treatment literature on how best to accomplish informed consent and organize this initial dialogue and intervention, among the most frequently cited are dialectical behavior therapy (DBT) (11), cognitive therapy for suicide prevention (CT-SP) (12), brief cognitive behavioral therapy for suicide prevention (BCBT-SP) (13, 14), attempted suicide short intervention program (ASSIP) (15), and the collaborative assessment and management of suicidality (CAMS) (16).

Despite differences in theoretical approaches and perspective, one of the identified commonalities of effective treatments is that they address the issue of individual responsibility in collaborative care as part of the broader informed consent process, doing so at the very beginning of care, and ultimately laying an important foundation for several other critical elements discussed below regarding planning for successful management of future episodes of suicidality and taking steps to reduce access to lethal means and improve safe storage. Additionally, they recognize that addressing the importance of a collaborative process and defining what that means in terms of patient behavior from the outset creates a unique opportunity to simultaneously target treatment hesitancy should it emerge (17). The most frequently used strategy to address treatment hesitancy among those that no-show for their first appointment is a brief phone call, allowing exploration and discussion of a range of issues, including (a) myths about treatment, (b) stigma and shame, (c) barriers to care, (d) that treatment is a time-limited process, and (e) a lack of hope regarding treatment effectiveness. It is important to note that any discussion targeting treatment hesitancy also creates an opportunity for development of a brief crisis management plan, including means safety steps, even if a decision is made to opt out of care. Specifically, discussion of the limited duration of treatment can be highly motivating [e.g., (4, 9–14) sessions of suicide-focused care] (16).

***Recommendation 1**: Clinicians engage the suicidal individual (and potentially key supporters) in discussion of anticipated risks of both pursuing clinical care and opting out of care, emphasizing that active clinical care has been demonstrated to reduce future risks and improve self-management skills.*

***Recommendation 2**: Clinicians clearly and specifically state that treatment is a collaborative endeavor, there is individual responsibility for active engagement in care, and that it helps to be proactive in identifying barriers to treatment adherence. As part of this discussion, clinicians specifically identify and discuss individual expectations for care such as: keeping scheduled appointments (or notifying when unable to attend), completing treatment exercises, developing a plan for management of future suicidal episodes, experimenting with new behaviors, sharing feelings (negative and positive), taking medications as prescribed, and limiting access to lethal means.*

***Recommendation 3:** When patients no show for their initial appointment, clinicians proactively address treatment hesitancy, with a brief phone call being the most widely used strategy to target myths about treatment and barriers to care. If the suicidal individual opts out of care, the clinician attempts to implement a plan to manage future suicidal crises and carefully documents this information.*

## Use of Explanatory Model That Targets Individual Self-Management Skills and Reduces Shame

One of the commonalties of treatments that work identified by Rudd and Munoz-Perez (4) emphasized the importance of an explanatory model targeting suicidal ideation and behaviors as transdiagnostic in nature and focused on the importance of self-awareness and emotional management skills, problem-solving, and active coping rather than describing suicidal ideation and behaviors as the direct result of core psychopathology, clinical diagnosis, and/or mental illness [e.g., (17)]. Again, reviewing and agreeing with the patient on an explanatory model to guide treatment is a clinical intervention, one that includes the elements summarized below as a framework for clinical content, and one that is frequently revisited and updated throughout care. Why use of such a model is effective is unclear at this point, but one possibility is that disconnecting suicidal ideation and behaviors from mental illness and psychiatric diagnosis may reduce stigma and shame common among those struggling with thoughts of killing themselves (18). Another possibility is that the core mechanisms underlying suicidal ideation and behaviors are transdiagnostic and therefore orthogonal to mental illness (19).

Although the majority of RCT’s that have been effective in reducing suicidal thinking and/or suicide attempts employ models often described or labeled as cognitive-behavioral in orientation, the underlying principles and clinical strategies used in these treatments are not unique to this single theoretical perspective. As noted above, examples of frequently cited treatment approaches utilized in RCT’s are Linehan’s (11) dialectical behavior therapy, Jobes’ CAMS (16, 20), CBT-SP (12), brief cognitive behavioral therapy (13, 14, 21), and Attempted Suicide Short Intervention Program (ASSIP) (15). Most (arguably all) of these treatment approaches employ interventions and strategies that target self-regulatory processes, especially strategies designed to improve the patient’s ability to recognize and change aversive and dysregulated emotional states. Relaxation skills training, for example, is frequently used to reduce episodes of acute autonomic arousal, which increases vulnerability to suicidal behavior. Cognitive reappraisal skills training is another commonly used clinical strategy. Critically, the cognitive reappraisal skills training used in these treatments focus on thoughts and beliefs that contribute directly to acute suicidal episodes (e.g., hopelessness, perceived burdensomeness, and entrapment) rather than thoughts and beliefs that are associated with depression, anxiety, and other psychiatric conditions in general.

Translating suicidality as a function of limited individual self and emotional managements skills, problem-solving, or ineffective coping has a number of advantages, including: (a) it can be shared with a suicidal individual in a manner that fits with each unique individual history allowing someone to understand how developmental experiences (e.g., including previous trauma, family history, and genetic predisposition) create individual vulnerability and limit the opportunity to develop and refine much needed self and emotional management skills, (b) the model is easy to understand, remember, and apply to daily life experiences, (c) it creates an opportunity to address apprehension about treatment as being a function of limited self and emotional management skills, (d) it has been demonstrated to fuel hope and wish to live (22), (e) it emphasizes the importance of individual responsibility in self-management, (f) recognizes the importance of and explains the destructive role of shame and guilt (23), and (g) translates to simple, skill-based interventions that can be practiced in session and utilized in the plan for management of future suicidal episodes. In short, effective interventions help patients develop a working model of their own suicidality and the nature of their own upset, so that targeted interventions be used to more effectively self-manage suicidal impulses.

It is, arguably, easiest to employ a multi-stage model when delivering care and reviewing the patient’s most recent suicidal episode. Common across the previously identified treatment approaches is clinical content including a recognition of multiple elements that provide the suicidal individual with an explanatory model, including individual history that creates potential vulnerabilities; environmental and contextual factors that activate and/or sustain suicidal episodes; interactions among suicide-related cognitions, beliefs, and reasons for dying; individual emotional and physiological response; and associated coping behaviors that have proven ineffective to date. Consistent across these treatments is an emphasis on the importance of creating meaning and purpose in life, ultimately enhancing the value of living.

***Recommendation 4**: Clinicians use an explanatory model that targets the development of self and emotional management skills as essential to resolving suicidal thoughts and behaviors. Ideally, the model will proactively reduce shame and identify the role of developmental history and related experiences (e.g., trauma) in understanding why skill development opportunities were limited earlier in life, and identify new ways for strengthening the subjective value of living relative to dying.*

## Proactively Identifying Barriers to Care and Problem Solving to Improve Treatment Adherence

Using defined clinical strategies that proactively intervene and engage the suicidal individual about treatment adherence and a focus on overcoming barriers to care is one of the common elements of treatments that work for suicidality. For the majority of treatments or interventions demonstrated to be effective, addressing the issue of treatment adherence is essential to the theoretical orientation and woven into the fabric of the conceptual model driving clinical care (24). Anticipating, identifying and effectively responding to treatment adherence problems recognizes that there are many reasons why someone might not be able to fully engage in treatment. Among strategies demonstrated effective are: (a) active discussion about barriers to care starting in the initial session, (b) a perspective that barriers to care are to be expected and understandable given the patient’s often limited resources, life context and limited self and emotional management skills, (c) recognition that poor treatment adherence is often not a conscious individual choice but the function of a convergence of multiple, difficult circumstances driving suicidality, (d) identification and discussion of the role of stigma, and (e) proactive assessment of the individual’s subjective belief they can complete the assigned task(s) (25). Within CAMS, for example, identifying potential barriers to treatment is a key element at the end of the CAMS Stabilization Plan—an essential tool used across the course of care (16). In BCBT, by comparison, the Commitment to Treatment Statement can be used to identify and problem solve a patient’s barriers to treatment (14, 26).

***Recommendation 5:** Clinicians anticipate, intervene, and proactively target treatment adherence difficulty as an expected and understandable challenge with suicidal individuals.*

## A Specific Plan for Management of Future Suicidal Episodes

One of the strongest points of convergence across RCTs is data regarding the effectiveness of safety planning, crisis response planning, and stabilization planning as clinical strategies that anticipate the emergence and importance of individual management of future suicidal episodes. Stanley et al. and Ferguson et al. (27, 28) have reported on the effectiveness of the safety planning intervention (SPI) for reducing suicidal behaviors, Tyndal et al. (29) have reported on the effectiveness of the CAMS stabilization plan for reducing suicidal ideation, and Bryan et al. (22) have reported on the effectiveness of the crisis response plan for reducing both suicidal behaviors and suicidal ideation. Despite differences in terminology and format (e.g., use of form templates vs. index cards or digital applications), the clinical strategies referenced above all target the development and implementation of a proactive and specific plan for individual management of future suicidal episodes. The specific steps used in each also vary somewhat but overlap substantially with respect to their mutual emphasis on (a) recognizing the onset of acute crises and/or having clearly articulated thresholds for use, often referred to as “warning signs”; (b) self-management activities (i.e., including a range of emotional regulation, self-soothing strategies, and distraction techniques; (c) thoughtful and careful use of social support; (d) steps for contacting crisis services and/or other sources of professional intervention (e.g., mental health professional’s phone number; National Suicide Prevention Lifeline for those in the U.S. or other crisis hotlines specific to the country); and (e) specific steps to follow to activate emergency service in the event self-management and other strategies prove ineffective.

***Recommendation 6:** Clinicians develop and implement a specific plan for the management of future acute suicidal episodes. The plan should include, at a minimum, the following identifiable steps: a) statement of threshold for use (i.e., when to use the plan), b) steps for individual self-management prior to any external intervention, c) use of external crisis phone intervention prior to having the suicidal individual go to the emergency room (e.g., National Suicide Prevention Lifeline, National Crisis Textline, national crisis line specific to the country, crisis lines for local service provider), d) a recommendation to go to a specific emergency room (provide address) if suicidality and motivation to die persists despite implementation of the previous steps.*

## A Specific Plan for Safe Storage and Limiting Access to Lethal Means

In light of a growing body of evidence demonstrating that availability of lethal means is strongly associated with suicide attempt lethality (7, 30, 31), that a large percentage of suicidal individuals experience significant and regular fluctuations in suicidal thinking and motivation to die (32), and that the decision to make a suicide attempt often occurs with very limited planning and within minutes to an hour of onset (33, 34), taking steps to limit access to lethal means is an important part of the broader clinical management strategy for suicidal individuals. Where a suicide attempt method is sufficiently common and sufficiently lethal, restricting or limiting access to the method significantly reduces suicide mortality (35).

Although mental health professionals often are not able to directly limit or restrict their patients’ access to potentially lethal methods for suicide, they can encourage and support means restriction via lethal means counseling. Across multiple RCTs, lethal means counseling has been shown to result in sustained improvement in environmental safety when delivered to the at-risk individual or the parents of at-risk youths (36). In the United States, owing to the extremely high case fatality rate of firearms when used as a suicide attempt method, clinicians are encouraged to ask about firearm access specifically, even with patients whose suicidal ideation and plans involve non-firearm methods. Anestis et al. (36) convincingly demonstrated that lethal means counseling and distribution of cable locks significantly increased the use of secure storage practices (e.g., storing ammunition separate from firearms, use of locking devices).

A key component of lethal means counseling is the development of a concrete plan to limit or reduce access to a given method. A common approach for developing these means safety plans is to include them as a component of the crisis management plans described in the previous section. The SPI and CAMS stabilization plan, for example, include a section focused on environmental safety. Alternatively, lethal means counseling can be conducted as a standalone intervention. A means safety plan, for example, can be developed in conjunction with crisis response planning. In BCBT, patients are invited to include family members and/or other trusted members of a patient’s social support network to assist with developing and implementing means safety plans and, often times, complete a “means safety receipt” (14, 37). Lethal means counseling could also be integrated into the informed consent process, potentially during discussions about treatment risks and benefits (4). Regardless of the specific approach employed to encourage or promote reduced access to potentially lethal suicide attempt methods, accumulating data support the effectiveness of lethal means counseling as a critical element of clinical care for suicidal individuals.

Consistent with injury prevention models, means safety plans that include procedures and steps for completely removing a potentially lethal method is probably most effective. However, complete removal of a given method is frequently not possible, whether due to practical limitations (e.g., removing all ligatures and sharp objects from a home) or patient preference (e.g., discomfort with surrendering one’s firearms, common among veterans). Under these circumstances, means safety plans can instead focus on placing barriers between patients and potentially lethal methods for suicide, thereby reducing or delaying immediate access to the method during an acute suicidal episode. Emerging evidence suggests that such barriers may serve to reduce the frequency and/or intensity of suicidal ideation (22, 38, 39), perhaps because these barriers serve as both physical psychological deterrents.

***Recommendation 7:** Regardless of methods identified by the suicidal individual, clinicians always inquire about access to and/or possession of a firearm or other potentially lethal means.*

***Recommendation 8:** Clinicians develop a specific plan to limit access to lethal means, including safe storage of firearms when present.*

## Informing the Public Health Narrative for Suicide Prevention

The distillation of available findings from RCTs targeting suicidality offer some simple but important recommendations for utilization of a collection of clinical strategies as part of a standard protocol for the delivery of care with suicidal individuals. Similarly, available data have important implications for the clinical suicide prevention narrative for suicide prevention. These data can be translated into several simple recommendations. First, suicide prevention campaigns should help reduce shame and stigma, move away from mental illness as a central explanatory variable, and emphasize that learning effective self and emotional management skills is essential to navigating life challenges and stressors, coupled with recognition that in many cases early trauma and adverse childhood experiences (ACEs) inhibit opportunities for much needed skill development. Mental illness is not the causal problem, rather, it is the individual’s ability to effectively manage the illness, coupled with access to resources that facilitate more effective self-management. Second, clinical suicide prevention narratives need to address the issue of barriers to effective care, acknowledging that these barriers are often part of the very stressors involved in elevating suicide risk, and actively work to remove these barriers and expand access. At the institutional or system level, this translates to recognition and acknowledgement of policies and practices that may well undercut access to effective care options. Third, clinical suicide prevention narratives can help undercut myths about clinical care and treatment that fuel treatment hesitancy, helping make it clear that life problems are solvable and emotional pain manageable. Fourth, the clinical suicide prevention narrative can share widely the importance of plans to manage future suicidal episodes, much like is the case with heart attack and stroke, offering specific recommendations for suicidal individuals to follow. Those plans need to include more than just the National Suicide Prevention Lifeline, Textline, or other country-specific crisis lines, expanding in thoughtful ways to local resources. And finally, the clinical suicide prevention narrative needs to embrace and expand the importance of safe storage of firearms and other lethal means as central to success in reducing the number of deaths by suicide.

RCT’s represent a unique resource for the delivery of clinical care to those struggling with suicidality. The current recommendations are a starting point for day-to-day clinical practice, recommendations that will need to be periodically revisited, revised, and updated as the scientific literature continues to grow. Additionally, incremental progress in the scientific foundation for clinical suicidology is essential to broader efforts in clinical suicide prevention. The identification of a common clinical strategies helps move this process forward with a small, but important step. One of the most significant advantages of a standard protocol is narrowing the broad variations in care, offering an empirically supported and clearly identifiable foundation for clinical care, one that reaches across the full range of clinical contexts. Ultimately, this may well help improve the targeted allocation of limited resources, with a net positive impact on the overall quality of care delivered.

A much needed and natural extension of these recommendations is identification and testing of criteria to assess adherence and fidelity of clinical delivery for the clinical strategies summarized. Subsequent work will offer a clinical toolkit for clinicians, coupled with applicable fidelity checklists for clinical delivery of care.

## Author Contributions

MD, CB, DJ, SF, and DC were involved in the conceptualization, development, and writing of the current manuscript. All authors contributed to the article and approved the submitted version.

## Conflict of Interest

DJ is an owner in CAMS-care. MD and SF own an interest in Oui Therapeutics. CB serves as a consultant for Oui Therapeutics. The remaining author declares that the research was conducted in the absence of any commercial or financial relationships that could be construed as a potential conflict of interest.

## Publisher’s Note

All claims expressed in this article are solely those of the authors and do not necessarily represent those of their affiliated organizations, or those of the publisher, the editors and the reviewers. Any product that may be evaluated in this article, or claim that may be made by its manufacturer, is not guaranteed or endorsed by the publisher.
